# A Rare Case of Idiopathic Angioedema Associated With Amlodipine Use

**DOI:** 10.7759/cureus.87661

**Published:** 2025-07-10

**Authors:** Nicole Kent, Kishan K Allikesam, Austin Ly, Melanie Veerasammy, Sai K Avula

**Affiliations:** 1 Medicine, Alabama College of Osteopathic Medicine, Dothan, USA; 2 Internal Medicine, Sri Venkateswara Medical College, Tirupati, IND; 3 Medicine, Southeast Health Medical Center, Dothan, USA; 4 Internal Medicine, Dothan Medical Associates, Dothan, USA

**Keywords:** acquired angioedema, calcium channel blockers, drug-induced angioedema, facial angioedema, medication therapy management

## Abstract

Angioedema is a self-limited, non-pitting edema of subcutaneous or submucosal tissues often affecting the face, lips, oral cavity, and airway. It may be histamine or bradykinin-mediated and is most commonly triggered by allergic reactions, medications, or systemic conditions. Drug-induced angioedema is frequently associated with angiotensin-converting enzyme (ACE) inhibitors, whereas calcium channel blockers (CCBs) like amlodipine are rarely implicated. This case report describes a rare case of recurrent angioedema in a hypertensive patient, ultimately attributed to amlodipine, and underscores the importance of a thorough evaluation and medication review in persistent idiopathic angioedema.

A 63-year-old male patient with a history of hypertension, hyperlipidemia, and obstructive sleep apnea presented with recurrent episodes of tongue swelling and dyspnea beginning one week after the initiation of ramipril and amlodipine. Ramipril was initially suspected and discontinued, yet symptoms persisted despite multiple antihypertensive regimen changes and negative evaluations for hereditary angioedema and carcinoid syndrome. Allergy testing indicated idiopathic histaminergic angioedema, and a variety of symptomatic treatments provided limited relief. Ultimately, discontinuation of amlodipine in November 2024 resulted in the complete resolution of angioedema, with no recurrence noted over the next few months of subsequent follow-up. The Naranjo Algorithm is a structured, 10-question tool used to determine the likelihood that an adverse drug reaction (ADR) is actually caused by a specific medication. It assesses factors such as the timing of the reaction, alternative causes, drug levels, and response to dechallenge or rechallenge, which determines if an ADR is definite, probable, possible, or doubtful. Retrospective assessment using this algorithm yielded a score of 7, indicating a probable ADR. This case highlights amlodipine as a rare but important cause of drug-induced angioedema. It emphasizes the need for careful medication review in patients with unexplained, recurrent angioedema and the benefit of considering less common etiologies when standard evaluations are inconclusive. Prompt recognition and discontinuation of the offending agent can result in full resolution and prevent unnecessary morbidity.

## Introduction

Angioedema is characterized by non-pitting edema in the subcutaneous or submucosal tissues, most commonly affecting the face, lips, neck, oral cavity, and extremities. It typically presents abruptly, with episodes that recur unpredictably and last between two and five days. The condition is usually mediated through histamine or bradykinin pathways and can be triggered instantly by allergic reactions, medications, or underlying systemic conditions [1].

Angioedema can be classified into two main types based on its underlying cause: hereditary angioedema, which results from a genetic deficiency in C1 esterase inhibitor, and drug-induced angioedema, which occurs as a side effect of various medications. One of the most commonly implicated drug classes is angiotensin-converting enzyme (ACE) inhibitors (ACEIs), which interfere with bradykinin degradation, leading to increased vascular permeability and subsequent tissue swelling [2]. Although rare, calcium channel blockers (CCBs), such as amlodipine, have also been linked to angioedema, with several case reports suggesting a potential association [3,4]. Less commonly, DPP-4 inhibitors used in managing type 2 diabetes, as well as aspirin and other non-steroidal anti-inflammatory drugs (NSAIDs), have been infrequently reported as triggers of the condition.

This report details a rare case of a 63-year-old male patient who developed angioedema after being prescribed amlodipine for hypertension. Following an extensive evaluation, it was determined that amlodipine was the likely cause of his condition. This case highlights the importance of thorough evaluation and personalized treatment adjustments to effectively address persistent angioedema.

## Case presentation

In March 2023, a 63-year-old male patient with a medical history of essential hypertension, hyperlipidemia, obstructive sleep apnea, and hypogonadism presented with new-onset shortness of breath and tongue swelling. He had been previously managed with hydrochlorothiazide (HCTZ) 25 mg for hypertension, but discontinued the medication after developing cramps due to an electrolyte imbalance. His primary care provider subsequently initiated Altace (ramipril) 10 mg and Norvasc (amlodipine) 5 mg to better control his blood pressure. One week after the initiation of Altace, the patient began experiencing shortness of breath and tongue swelling.

Upon evaluation, the patient had localized non-pitting edema to his lips and tongue, intact skin with no discoloration, and no urticaria. He had been experiencing dyspnea and wheezing. Altace was discontinued and switched to Coreg (carvedilol) 6.25 mg, to be taken twice daily. HCTZ was transitioned to chlorthalidone 25 mg once daily. Albuterol sulfate was also prescribed for his dyspnea and wheezing.

To evaluate for hereditary angioedema due to C1 esterase inhibitor, C1 esterase inhibitor and C4 complement levels were ordered, but they were within normal limits, effectively ruling this out. Additionally, 5-hydroxyindoleacetic acid (5-HIAA) levels were checked to investigate the possibility of a carcinoid tumor, but the results were negative. The patient’s wheezing and dyspnea had resolved between April and June 2023.

In June 2023, the patient returned with a recurrence of angioedema affecting the tongue and reported that these episodes had become more frequent, occurring 2-3 times a month. The patient noted that taking Allegra (fexofenadine) 180 mg as needed provided limited relief, but no clear pattern of occurrence could be identified. He denied any recent changes in diet, activity, environmental exposure, or any known allergies. Given the persistence of symptoms, allergy testing was performed, and the results were consistent with idiopathic histaminergic angioedema, which is defined as a condition of recurrent swelling without a known cause, triggered by histamine release, and typically responsive to antihistamines. Based on this, the primary care provider initiated hydroxyzine HCl 25 mg once daily for symptom control. After consulting with an allergy specialist, the Allegra dose was increased to two 180 mg tablets daily. Despite the absence of associated urticaria, diarrhea, vomiting, or flushing, the patient's symptoms suggested probable mast-cell activation, and a trial of omalizumab (Xolair) 300 mg every four weeks was considered. However, this option was not pursued due to insurance denial. The patient continued to experience episodes 2-3 times per month, managing them with Allegra, hydroxyzine, and albuterol, with limited relief.

In November 2024, due to ongoing episodes of tongue angioedema, a decision was made to discontinue amlodipine, given the rare but documented association between the medication and angioedema. The patient was switched back to HCTZ 12.5 mg once daily. Following the discontinuation of amlodipine, the patient’s angioedema completely resolved within a few days, and no further concerns have been reported as of his three-month follow-up. The patient is being monitored for electrolyte imbalances, with appropriate repletion as needed.

## Discussion

We report a rare case of amlodipine-induced angioedema in a 63-year-old man with long-standing hypertension treated with several commonly used antihypertensive agents. The diagnostic process highlights the complexity of identifying drug-induced angioedema, particularly in patients taking multiple agents that may influence the bradykinin or histamine pathways.

Due to the known association of ACEIs with angioedema, ramipril was initially suspected as the cause due to its known association with this adverse effect [1,3]. However, discontinuation of ramipril did not alleviate the patient’s symptoms. Further evaluation ruled out hereditary angioedema (normal C1 esterase inhibitor and C4 levels) and carcinoid syndrome (normal 5-HIAA levels), narrowing the differential diagnosis. Persistent episodes of angioedema prompted consideration of amlodipine as a possible etiology. Upon discontinuation of amlodipine, the patient experienced complete symptom resolution. The Naranjo Algorithm is a structured, 10-question tool used to determine the likelihood that an adverse drug reaction (ADR) is actually caused by a specific medication. It assesses factors such as the timing of the reaction, alternative causes, drug levels, and response to dechallenge or rechallenge, which determines if an ADR is definite, probable, possible, or doubtful. A Naranjo ADR probability score of 7 retrospectively supported amlodipine as a probable cause.

Although dihydropyridine CCBs such as amlodipine are not commonly associated with angioedema, there is increasing awareness of this rare adverse reaction [4]. CCBs are known to cause dermatologic reactions including pruritic eruptions and hypersensitivity, and isolated cases of amlodipine-induced angioedema have been documented, as referenced below. Typically, these cases involve facial or oropharyngeal swelling. In the present case, the patient experienced recurrent angioedema localized to the tongue without associated urticaria or systemic allergic manifestations, consistent with a non-IgE-mediated mechanism.

Both dihydropyridine and non-dihydropyridine CCBs have been implicated in angioedema. One article described three adult cases of CCB-induced angioedema resolving within 72 hours of drug discontinuation [5]. Pediatric cases have also been reported. Another article described a two-year-old with upper airway obstruction managed with dexamethasone and epinephrine [2], and another report described persistent macroglossia in an eight-year-old following nicardipine and amlodipine administration, which resolved upon withdrawal of the offending agents [6].

The Antihypertensive and Lipid-Lowering Treatment to Prevent Heart Attack Trial (ALLHAT) offers the most comprehensive epidemiologic estimate, reporting an incidence of 0.03% for amlodipine-induced angioedema [3]. Despite this, the condition remains under-recognized, likely due to its rarity and delayed onset compared to ACEI-induced angioedema. Furthermore, the literature lacks detailed characterization of onset timing, dose-response relationships, or predisposing risk factors.

This case underscores the challenges of diagnosing rare ADRs in outpatient settings, particularly when symptoms are intermittent and non-life-threatening. The diagnostic process spanned approximately 20 months, due to the lack of systemic features and subtle presentation. Nonetheless, this case illustrates the importance of comprehensive medication review and the need for maintaining a broad differential diagnosis when managing unexplained, recurrent angioedema.

The patient's complete symptom resolution following amlodipine discontinuation emphasizes the diagnostic value of drug withdrawal. While treatment of angioedema may include corticosteroids, antihistamines, or epinephrine in more severe cases, management should be guided by severity and presumed mechanism. Additional research is warranted to define optimal treatment protocols and identify risk factors for CCB-induced angioedema.

In conclusion, clinicians should consider amlodipine as a potential etiology in cases of recurrent angioedema when more common causes have been excluded. Ongoing medication reassessment and individualized patient evaluation remain essential to timely diagnosis and appropriate management.

## Conclusions

This case highlights the importance of considering medication-induced angioedema in the differential diagnosis of recurrent angioedema. Although amlodipine is rarely associated with this adverse effect, it was identified as the likely cause in this patient after a thorough evaluation and medication review. Timely recognition and discontinuation of the offending agent resulted in complete symptom resolution and prevented further complications. Clinicians should maintain a high index of suspicion for less common drug-related etiologies in patients presenting with recurrent angioedema, especially when standard causes have been ruled out. Comprehensive medication reconciliation and ongoing monitoring are essential to ensure optimal patient outcomes.
